# Sex Differences in Brain Region-Specific Activation of c-Fos following Kappa Opioid Receptor Stimulation or Acute Stress in Mice

**DOI:** 10.3390/ijms242015098

**Published:** 2023-10-11

**Authors:** Qianhan Ma, Susan Wonnacott, Sarah J. Bailey, Christopher P. Bailey

**Affiliations:** Department of Life Sciences, University of Bath, Bath BA2 7AY, UK

**Keywords:** kappa opioid receptor, U50,488, norBNI, forced swim stress, depression, addiction, nucleus accumbens, prefrontal cortex, amygdala

## Abstract

Kappa opioid receptors (KOPr) are involved in the response to stress. KOPr are also targets for the treatment of stress-related psychiatric disorders including depression, anxiety, and addiction although effects of KOPr are often sex-dependent. Here we investigated c-Fos expression in a range of brain regions in male and female mice following an acute stressor, and a single injection of KOPr agonist. Using adult C57BL/6 c-Fos-GFP transgenic mice and quantitative fluorescence microscopy, we identified brain regions activated in response to a challenge with the KOPr agonist U50,488 (20 mg/kg) or an acute stress (15 min forced swim stress, FSS). In male mice, U50,488 increased expression of c-Fos in the prelimbic area of the prefrontal cortex (PFCx), nucleus accumbens (NAcc), and basolateral nuclei of the amygdala (BLA). In contrast, in female mice U50,488 only activated the BLA but not the PFCx or the NAcc. FSS increased activation of PFCx, NAcc, and BLA in males while there was no activation of the PFCx in female mice. In both sexes, the KOPr antagonist norBNI significantly blocked U50,488-induced, but not stress-induced activation of brain regions. In separate experiments, activated cells were confirmed as non-GABAergic neurons in the PFCx and NAcc. Together these data demonstrate sex differences in activation of brain regions that are key components of the ‘reward’ circuitry. These differential responses may contribute to sex differences in stress-related psychiatric disorders and in the treatment of depression, anxiety, and addiction.

## 1. Introduction

Stress is a major risk factor for developing a number of psychiatric conditions including depression, anxiety, and substance misuse [1,2]. The acute stress response involves perception of physical or emotional stress and results in activation of the hypothalamic–pituitary–adrenal (HPA) axis and the secretion of glucocorticoids. While the paraventricular nucleus in the hypothalamus may represent the final common pathway for the integration of stress responses in the brain, there is a complex neurocircuitry involved in the adaptive behavioural response to chronic stress [3]. The paraventricular nucleus is directly excited by afferents from the brainstem and hypothalamic circuits. Human functional imaging has identified multiple brain areas that are activated during stress, including the prefrontal cortex (PFCx), amygdala, hypothalamus, and nuclei of the brain stem [4,5]. Additionally, there is mounting evidence of the involvement of reward processing areas such as the nucleus accumbens (NAcc) in stress-responsiveness [6].

The kappa opioid receptor (KOPr), and its endogenous ligand dynorphin, are highly expressed in the brain regions involved in responding to stress [7]. Imaging studies have also shown altered activity in the amygdala, hippocampus, NAcc, and PFCx of psychiatric patients [8]. The dysphoric component of stress has been suggested to be mediated by KOPr [9]. Dynorphin, released during stress exposure [10], or administration of KOPr agonists induces dysphoria in humans [11] and alters stress-responsive behaviours in rodents, for example in the forced swim test and conditioned-place aversion [12,13,14]. In contrast, KOPr gene deletion or prodynorphin gene disruption has the ability to block stress-induced behavioural adaptations [14,15]. The ability of KOPr antagonists, such as norbinaltorphimine (norBNI), to block stress-induced behaviours, and to have antidepressant- and anxiolytic-like effects in rodents, has made KOPr antagonists a target of drug development [16,17,18].

Sex differences are evident in psychiatric disorders and their treatment [19,20]. For example, one of the most consistent findings in psychiatry is that women have higher rates of depression and more anxiety disorders than men [20,21,22]. While men are more likely to have a drug abuse/dependence disorder, women are more vulnerable to rapid escalation of drug use and at greater risk for relapse following abstinence [23]. Stress is a major risk factor for these conditions and there are some important sex differences in the response to stress. In healthy human volunteers, stress-related HPA axis responses to psychological stress generally show a higher cortisol response in men than women, although some studies show no sex differences [24]. The mechanisms of how sex differences in stress-responsiveness could contribute to sex differences in psychiatric disease are not clearly delineated.

Numerous studies have demonstrated sex differences in KOPr function, in terms of antinociceptive, dysphoric, and stress-related behaviours [25]. Early studies in deer mice showed that administration of U50,488, a KOPr agonist, produced significantly greater analgesia in male, than in female mice [26]. Liu and colleagues showed that intrathecal administration of the adrenoceptor antagonist yohimbine produced an anti-nociceptive effect by releasing dynorphin and activating the KOPr system in male rats, but not in females [27]. More recently, the relative lack of KOPr mediated analgesic effects in female mice has been shown to be oestrous cycle dependent [28,29]. Abraham et al. demonstrated that oestradiol in female mice increased GRK2 phosphorylation and reduced G-protein signalling leading to attenuated analgesic responses. These authors also suggested that while analgesic effects of KOPr activation may be regulated by gonadal hormones, the dysphoric effects are not, and revealed no significant differences in conditioned place aversion behaviour between males and females [28]. Other studies have demonstrated sex differences in KOPr agonist-induced dysphoria and negative affect. Female rats were significantly less sensitive than males to the threshold-increasing effects of U50,488 in an intracranial self-stimulation responding task that was independent of oestrous cycle stage [30] and conditioned place aversion induced by U50,488 was observed to be more robust in males than in females [31]. In mice, U50,488 showed dose-dependent differences in conditioned place aversion between females and males, with no sex differences in the anti-pruritic and hypo-locomotor effects [32]. Another study showed that female mice required a higher dose of U50,488 to suppress nest building behaviour, compared to males, an effect inhibited by the oestrogen receptor inhibitor tamoxifen [33]. Both stress and KOPr agonist increased activity of early growth response 1 (EGR1, an immediate early gene that is an indirect marker of neuronal activity) in the NAcc in female California mice, but not in males [34].

Understanding sex differences in stress-responsive neurocircuitry, and the role of KOPr, is critical to the development of novel opioid ligands and improving our understanding of female psychopharmacology [16,19]. Given the fundamental interplay between KOPr and stress, here we tested the hypothesis that there are sex differences in the neuronal circuitry activated by these stimuli. We measured the expression of the immediate early gene c-Fos, a marker of neuronal activation ex vivo, widely used to map neuronal changes in response to a range of stimuli [35]. c-Fos expression was examined in several key brain regions (PFCx, NAcc, hippocampus, and amygdala), in both male and female mice, in response to a physical stressor (acute forced swim stress, FSS) and a single administration of a KOPr agonist (U50,488).

## 2. Results

### 2.1. Effects of Systemic Administration of U50,488 on c-Fos Expression in the PFCx of Male and Female Mice

**Figure 1:**
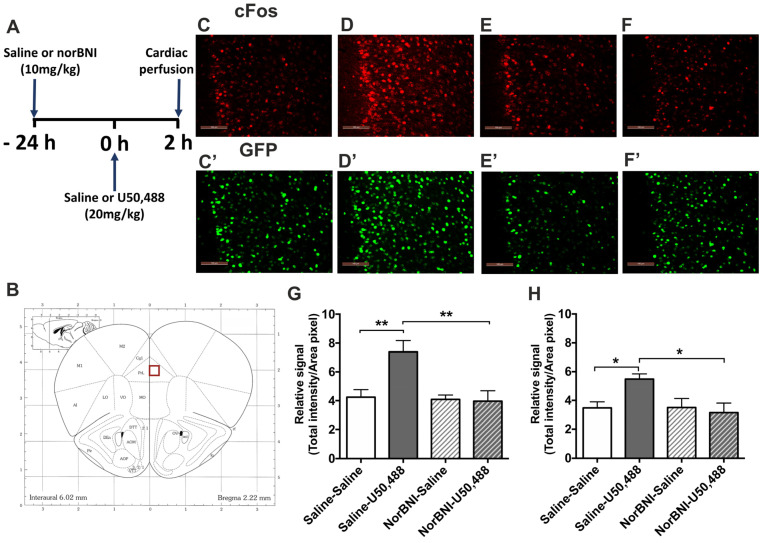
**The effect of U50,488 on c-Fos and c-Fos-driven GFP expression in the PFCx of male c-Fos-GFP transgenic mice.** (**A**) Experimental timeline showing administration of norBNI (10 mg/kg, ip), or saline, 24 h prior to KOPr activation with U50,488 (20 mg/kg, ip). (**B**) Schematic coronal section from mouse brain atlas [38] showing region analysed; red square indicates the prelimbic region of the prefrontal cortex. (**C**–**F**) Representative images showing immunolabelling for c-Fos (red, **C**–**F**) and GFP (green, **C’**–**F’**) following treatment with saline/saline (**C**,**C’**), saline/U50,488 (**D**,**D’**), norBNI/saline (**E**,**E’**), norBNI/ U50,488 (**F**,**F’**). Scale bar: 100 μm. (**G**,**H**) Quantification of immunolabelling for c-Fos (**G**), and c-Fos-driven GFP (**H**) expression. All data are represented as mean ± SEM (n = 6 animals/treatment group). One-way ANOVA with post hoc Sidak’s test * *p* < 0.05, ** *p* < 0.01.

**Figure 2:**
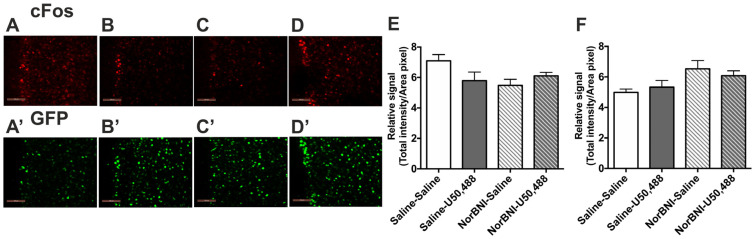
**The effect of U50,488 on c-Fos and c-Fos-driven GFP expression in the PFCx of female c-Fos-GFP transgenic mice.** (**A**–**D**) Representative images showing immunolabelling for c-Fos (**A**–**D**) and GFP (**A’**–**D’**) following treatment with saline/saline (**A**,**A’**), saline/U50,488 (**B**,**B’**), norBNI/saline (**C**,**C’**), norBNI/ U50,488 (**D**,**D’**). Scale bar: 100 μm. (**E**,**F**) Quantification of immunolabelling for c-Fos (**E**) and c-Fos-driven GFP (**F**) expression. All data are represented as mean ± SEM (n = 6 animals/treatment group).

The immediate early gene c-Fos was used to quantify the extent of brain region activation in response to KOPr activation. c-Fos-GFP transgenic mice were employed, enabling measurement of both c-Fos itself and c-Fos-driven GFP expression. The latter has been suggested to be more stable, with a relatively longer half-life, than c-Fos, hence these transgenic mice could show c-Fos-driven GFP expression at time-points after c-Fos expression has peaked [36,37]. The effect of the KOPr agonist U50,488 (20 mg/kg) in adult male c-Fos-GFP transgenic mice on the C57BL/6 background is shown in Figure 1. To demonstrate the selective involvement of KOPr in the agonist-induced response, the KOPr antagonist norBNI, or saline, was administered 24 h prior to U50,488 and one-way ANOVA followed by post hoc Sidak’s test used to test the effects of treatment (Figure 1A). Quantification of c-Fos immunolabelling revealed a significant effect of treatment (Figure 1G, *F*(3, 20) = 7.096, *p* = 0.002). U50,488 treatment significantly increased c-Fos labelling in the prelimbic area (layers 1–3) of the PFCx compared to saline-treated controls (*p* < 0.01, n = 6 per group). Analysis of c-Fos-driven GFP expression in these same tissue sections was also significantly increased following U50,488 administration (Figure 1H, *F*(3, 20) = 4.045, *p* = 0.021; pairwise comparison to saline *p* < 0.05). The KOPr antagonist norBNI, administered alone, was without significant effects on either c-Fos or c-Fos-driven GFP expression but was able to block U50,488-induced effects. Interestingly, when the same experiment was conducted in adult female mice, there was no significant effect of treatment on immunofluorescence for c-Fos (*F*(3, 20) = 2.779, *p* = 0.068) or c-Fos-driven GFP expression (*F*(3, 20) = 3.049, *p* = 0.052) in the PFCx (Figure 2).

### 2.2. Effects of Acute FSS on c-Fos Expression in the PFCx of Male and Female Mice

**Figure 3:**
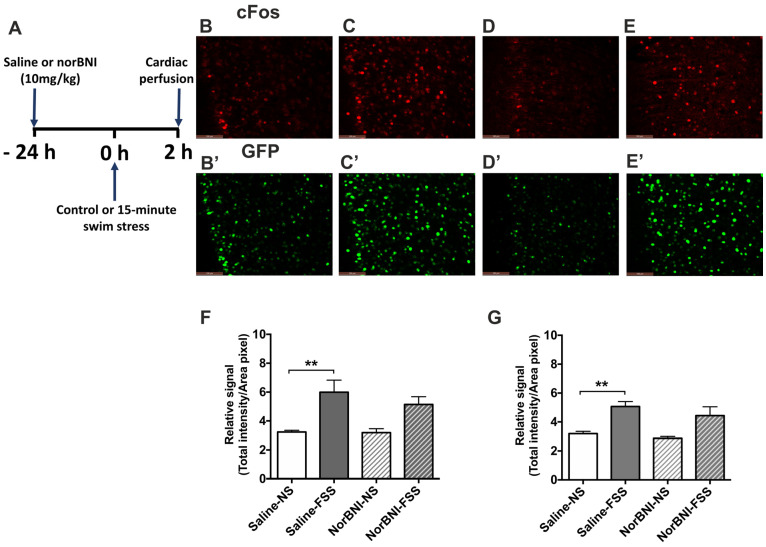
**The effect of acute FSS on c-Fos and c-Fos-driven GFP expression in the PFCx of male c-Fos-GFP transgenic mice.** (**A**) Experimental timeline showing administration of norBNI (10 mg/kg, ip), or saline, 24 h prior to 15 min FSS. (**B**–**E**) Representative images showing immunolabelling for c-Fos (**B**–**E**) and GFP (**B’**–**E’**) following treatment with saline/no stress (NS) controls (**B**,**B’**), saline/FSS (**C**,**C’**), norBNI/ no stress (NS) (**D**,**D’**), norBNI/ FSS (**E**,**E’**). Scale bar: 100 μm. (**F**,**G**) Quantification of immunolabelling for c-Fos (**F**), and c-Fos-driven GFP (**G**) expression. All data are represented as mean ± SEM (n = 6 animals/treatment group). One-way ANOVA with post hoc Sidak’s test ** *p* < 0.01.

**Figure 4:**
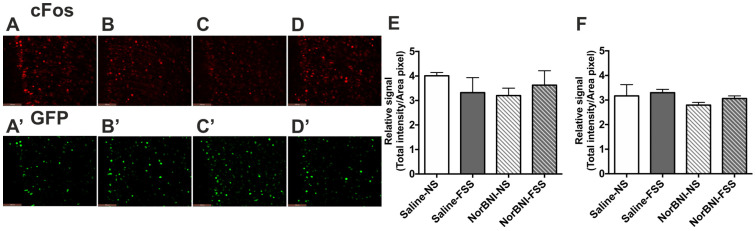
**The effect of acute FSS on c-Fos and c-Fos-driven GFP expression in the PFCx of female c-Fos-GFP transgenic mice.** (**A**–**D**) Representative images showing immunolabelling for c-Fos (**A**–**D**) and GFP (**A’**–**D’**) following treatment with saline/no stress (NS) controls (**A**,**A’**), saline/FSS (**B**,**B’**), norBNI/ no stress (NS) (**C**,**C’**), norBNI/ FSS (**D**,**D’**). Scale bar: 100 μm. (**E**,**F**) Quantification of immunolabelling for c-Fos (**E**), and c-Fos-driven GFP (**F**) expression. All data are represented as mean ± SEM (n = 6 animals/treatment group).

A physical stressor, an acute 15 min FSS, produced a similar pattern of activation in the PFCx of male mice as did KOPr activation; there was a significant increase in immunolabelling for both c-Fos (*F*(3, 20) = 7.335, *p* = 0.002) and c-Fos-driven GFP (*F*(3, 20) = 8.187, *p* = 0.001) expression following acute stress (Figure 3, pairwise comparison to saline *p* < 0.01, n = 6 per group). In the presence of norBNI, the acute stress did not cause a statistically significant increase in neuronal activation, but neither was the effect abolished (Figure 3G,H). In contrast, in female mice FSS had no significant effect on immunofluorescence for c-Fos (*F*(3, 20) = 3.785, *p* = 0.146) or c-Fos-driven GFP (*F*(3, 20) = 0.746, *p* = 0.537) expression in the PFCx (Figure 4).

### 2.3. Effects of Activating KOPr and Acute FSS on c-Fos Expression in the NAcc

**Figure 5:**
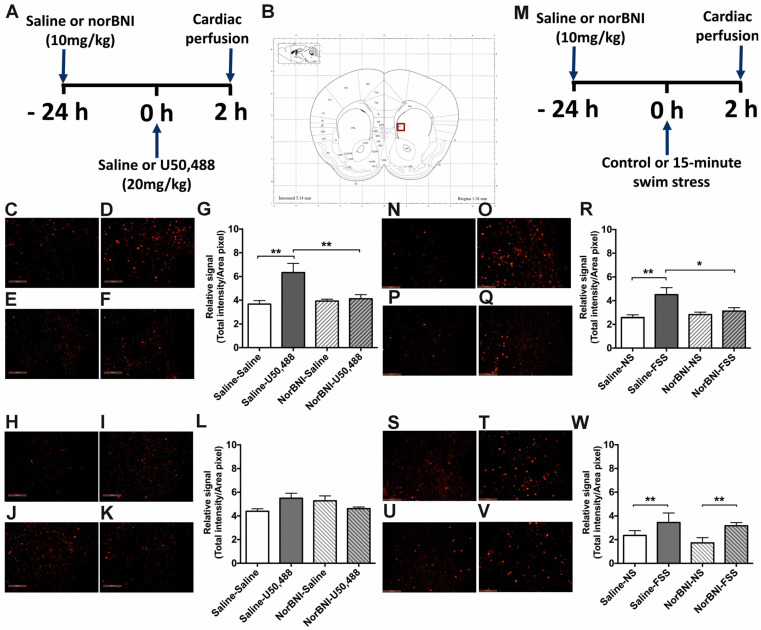
**The effect of U50,488 and FSS on c-Fos expression in the NAcc of male and female c-Fos-GFP transgenic mice.** (**A**) Experimental timeline showing administration of norBNI (10 mg/kg, ip), or saline, 24 h prior to KOPr activation with U50,488 (20 mg/kg, ip). (**B**) Schematic coronal section from mouse brain atlas [38] showing region analysed, red square indicates the NAcc core region. Representative images of male (**C**–**F**) and female (**H**–**K**) NAcc showing immunolabelling for c-Fos following treatment with saline/saline (**C**,**H**), saline/U50,488 (**D**,**I**), norBNI/saline (**E**,**J**), norBNI/ U50,488 (**F**,**K**). Scale bar: 100 μm for all images. Quantification of immunolabelling for c-Fos in male (**G**) and female (**L**) sections. (**M**) Experimental timeline showing administration of norBNI (10 mg/kg, ip), or saline, 24 h prior to 15 min FSS. Representative images of male (**N**–**Q**) and female (**S**–**V**) NAcc showing immunolabelling for c-Fos following treatment with saline/no stress (NS) controls (**N**,**S**), saline/FSS (**O**,**T**), norBNI/ no stress (NS) (**R**,**U**), norBNI/ FSS (**Q**,**V**). Quantification of immunolabelling for c-Fos in male (**S**) and female (**W**) sections. All data are represented as mean ± SEM (n = 6 animals/treatment group). One-way ANOVA with post hoc Sidak’s test * *p* < 0.05, ** *p* < 0.01.

We next investigated if the effects seen in male and female PFCx were consistent across a number of brain regions involved in stress-responsive neurocircuitry. In the NAcc of adult male mice, analysis of c-Fos immunolabelling revealed a significant effect of treatment (Figure 5G, *F*(3, 20) = 7.337, *p* = 0.002). U50,488 induced a significant increase in c-Fos immunolabelling, compared to saline-treated controls, that was abolished by pre-treatment with norBNI (*p* < 0.01, n = 6 per group). Data for c-Fos driven GFP expression are qualitatively similar (Supplementary Figure S2). Acute FSS also produced a significant increase in immunofluorescence for c-Fos in the NAcc of male mice. In contrast to the PFCx, this was also blocked by norBNI administration (Figure 5S, *F*(3, 20) = 5.887, *p* = 0.005; pairwise comparisons *p* < 0.01, n = 6). c-Fos-driven GFP signals produced a similar finding except that the blocking effect of norBNI was not observed (Supplementary Figure S2).

In the NAcc of adult female mice, there was no significant effect of U50,488 or norBNI treatment on immunofluorescence for c-Fos (Figure 5M, *F*(3, 20) = 2.66, *p* = 0.076) or c-Fos-driven GFP expression (Supplementary Figure S2). However, an acute FSS did produce a significant increase in c-Fos immunolabelling (Figure 5W, *F*(3, 20) = 13.62, *p* = 0.005, pairwise comparison to saline *p* < 0.01, n = 6 per group) and c-Fos-driven GFP expression (Supplementary Figure S2) in the NAcc. NorBNI had no significant effect (Figure 5W).

### 2.4. Effects of Activating KOPr and Acute FSS on c-Fos Expression in the Hippocampus

**Figure 6:**
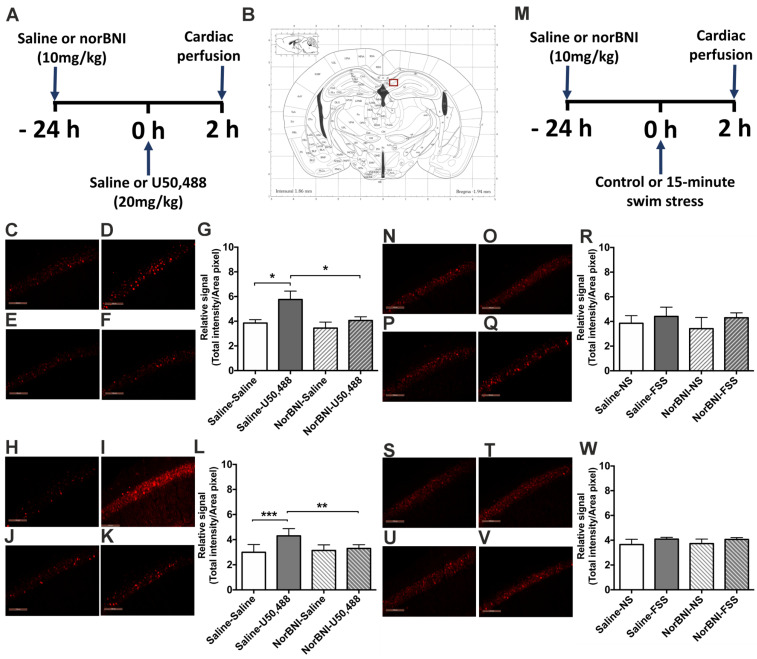
**The effect of U50,488 and FSS on c-Fos expression in the CA1 region of the hippocampus of male and female c-Fos-GFP transgenic mice.** (**A**) Experimental timeline showing administration of norBNI (10 mg/kg, ip), or saline, 24 h prior to KOPr activation with U50,488 (20 mg/kg, ip). (**B**) Schematic coronal section from mouse brain atlas [38] showing region analysed; red square indicates the hippocampus CA1 region. Representative images of male (**C**–**F**) and female (**H**–**K**) hippocampus showing immunolabelling for c-Fos following treatment with saline/saline (**C**,**H**), saline/U50,488 (**D**,**I**), norBNI/saline (**E**,**J**), norBNI/U50,488 (**F**,**K**). Scale bar: 100 μm for all images. Quantification of immunolabelling for c-Fos in male (**G**) and female (**L**) sections. (**M**) Experimental timeline showing administration of norBNI (10 mg/kg, ip), or saline, 24 h prior to 15 min FSS. Representative images of male (**N**–**Q**) and female (**S**–**V**) hippocampus showing immunolabelling for c-Fos following treatment with saline/no stress (NS) controls (**N**,**S**), saline/FSS (**O**,**T**), norBNI/no stress (NS) (**P**,**U**), norBNI/FSS (**Q**,**V**). Quantification of immunolabelling for c-Fos in male (**R**) and female (**W**) sections. All data are represented as mean ± SEM (n = 6 animals/treatment group). One-way ANOVA with post hoc Sidak’s test * *p* < 0.05, ** *p* < 0.01, *** *p* < 0.001.

In the hippocampus we investigated neuronal activation in both the CA1 and dentate gyrus subregions. In both male and female adult mice, U50,488 significantly increased c-Fos immunolabelling in the CA1 region and that was blocked by pre-administration of norBNI, indicative of KOPr activation (Figure 6G, *F*(3, 20) = 4.837, *p* = 0.011, pairwise comparison *p* < 0.05; Figure 6M, *F*(3, 20) = 8.472, *p* = 0.0008, pairwise comparison *p* < 0.001, n = 6 per group). U50,488 also induced significant c-Fos-driven GFP expression in hippocampal CA1 region of male, but not female, adult mice (Supplementary Figure S3). In contrast, an acute FSS had no effect on CA1 immunofluorescence for c-Fos in both male and female mice (Figure 6S, *F*(3, 20) = 2.588, *p* = 0.082; Figure 6W, *F*(3, 20) = 3.444, *p* = 0.057) or on CA1 c-Fos-driven GFP expression (Supplementary Figure S3). In the dentate gyrus, in both male and female mice, c-Fos immunofluorescence and c-Fos driven GFP expression were not significantly altered by either U50,488 injection or acute FSS (Supplementary Figures S4 and S5). Overall, in the hippocampus similar c-Fos expression patterns were observed in both male and female mice. Within the hippocampus neither the dentate gyrus nor the CA1 was activated by FSS whereas only the CA1 was activated by KOPr agonist stimulation.

### 2.5. Effects of Activating KOPr and Acute FSS on c-Fos Expression in the Amygdala

**Figure 7:**
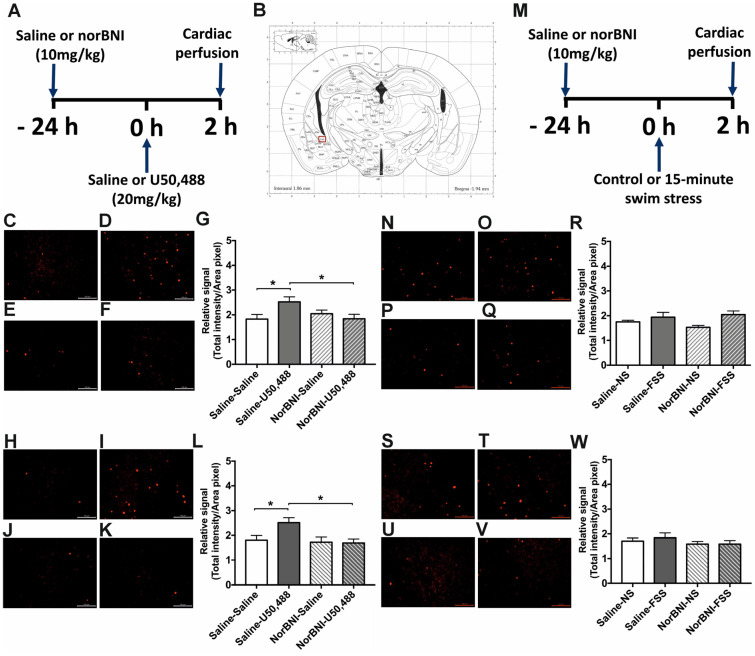
**The effect of U50,488 and FSS on c-Fos expression in the CeA of male and female c-Fos-GFP transgenic mice.** (**A**) Experimental timeline showing administration of norBNI (10 mg/kg, ip), or saline, 24 h prior to KOPr activation with U50,488 (20 mg/kg, ip). (**B**) Schematic coronal section from mouse brain atlas [38] showing region analysed; red square indicates the CeA. Representative images of CeA from male (**C**–**F**) and female mice (**H**–**K**), showing immunolabelling for c-Fos following treatment with saline/saline (**C**,**H**), saline/U50,488 (**D**,**I**), norBNI/saline (**E**,**J**), norBNI/U50,488 (**F**,**K**). Scale bar: 100 μm for all images. Quantification of immunolabelling for c-Fos in male (**G**) and female (**L**) sections. (**M**) Experimental timeline showing administration of norBNI (10 mg/kg, ip), FSS or saline, 24 h prior to 15 min FSS. Representative images of CeA from male (**N**–**Q**) and female mice (**S**–**V**) showing immunolabelling for c-Fos following treatment with saline/no stress (NS) controls (**N**,**S**), saline/FSS (**O**,**T**), norBNI/no stress (NS) (**P**,**U**), norBNI/FSS (**Q**,**V**). Quantification of immunolabelling for c-Fos in male (**R**) and female (**W**) sections. All data are represented as mean ± SEM (n = 6 animals/treatment group). One-way ANOVA with post hoc Sidak’s test * *p* < 0.05.

In the amygdala we investigated neuronal activation in both the basolateral nucleus (BLA) and central nucleus (CeA) subregions. In both male and female adult mice, U50,488 significantly increased c-Fos immunolabelling in the CeA that was blocked by pre-administration of norBNI (Figure 7G, *F*(3, 20) = 3.119, *p* = 0.049; Figure 7M, *F*(3, 20) = 4.269, *p* = 0.018, pairwise comparisons *p* < 0.05, n = 6 per group). U50,488 induced similar effects in c-Fos-driven GFP expression in the same region (Supplementary Figure S6). In contrast, in both male and female adult mice, an acute FSS was without any significant effects on immunofluorescence for c-Fos (Figure 7S, *F*(3, 20) = 17.51, *p* = 0.062; Figure 7W, *F*(3, 20) = 0.686, *p* = 0.571) or c-Fos-driven GFP expression (Supplementary Figure S6). In the BLA, in both male and female mice, both U50,488 and acute FSS induced significant increases in c-Fos and c-Fos-driven GFP expression (Supplementary Figures S7 and S8). Pre-administration of norBNI abolished the effects of U50,488 administration but not the FSS-induced increase in neuronal activation (Supplementary Figures S7 and S8). Overall, in the amygdala, similar c-Fos expression patterns were observed in both male and female mice. Within the amygdala the BLA was activated by swim stress whereas the CeA was not but both regions were activated by KOPr agonist stimulation.

### 2.6. KOPr- and Acute FSS-Activated Cells Are Predominantly Non-GABAergic Neurons

**Figure 8:**
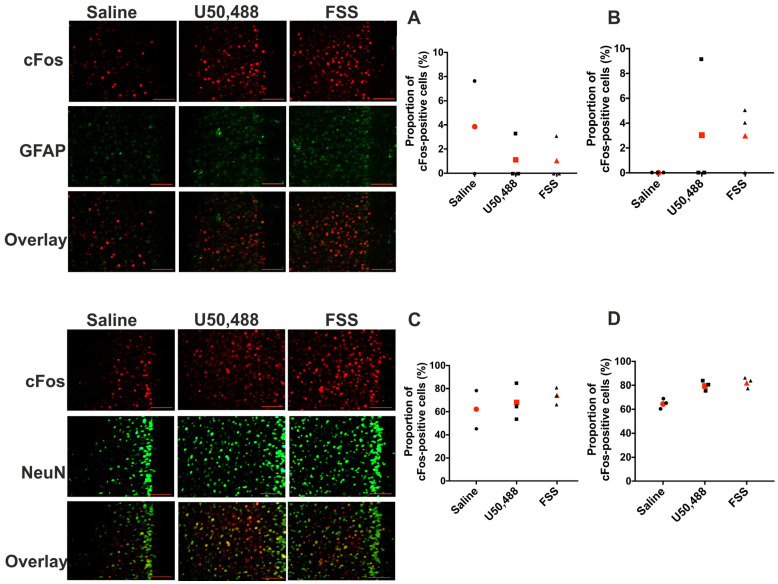
Activated c-Fos-expressing cells in the PFCx of male and female c-Fos-GFP transgenic mice are predominantly neuronal cells. Representative images of PFCx from male mice showing immunolabelling for c-Fos, glial fibrillary acidic protein (GFAP) and the neuronal nuclear protein NeuN following U50,488 (20 mg/kg, ip) treatment or 15 min FSS. Quantification of the proportion of c-Fos positive cells that are double labelled for GFAP (**A**,**B**) or for Neu N (**C**,**D**) in male (**A**,**C**) and female (**B**,**D**) sections. Data for each individual sample are shown (black symbols) and the mean (red symbols), n = 2–3 animals/treatment group. Scale bar: 100 μm for all images.

c-Fos is not only expressed in neurons but also in glial cells [39]. Using double immunolabeling with the neuronal marker NeuN (neuronal nuclear protein) and the glial marker GFAP (glial fibrillary acidic protein), in a separate series of experiments, we confirmed that the stimuli used in our study induce c-Fos expression in neuronal cells (Figure 8). In the PFCx of both male and female mice, the proportion of c-Fos positive cells that also expressed GFAP was ≤3% in both the U50,488 and acute stress-treated groups (Figure 8A,B). For example, in 6 stress-treated animals (3× male; 3× female), 203 neurons were c-Fos positive, 3 of which were also positive for GFAP. In these same animals, the proportion of c-Fos positive cells that also expressed NeuN was 68% (male) and 79% (female) in U50,488 treatment groups, 74% (male) and 81% (female) in acute stress treatment groups (Figure 8C,D). Comparable data for c-Fos expression in the NAcc are qualitatively similar (Supplementary Figure S9). Taken together these data show that the vast majority of cells activated by both U50,488 and FSS, in the PFCx and NAcc, are neuronal.

**Figure 9:**
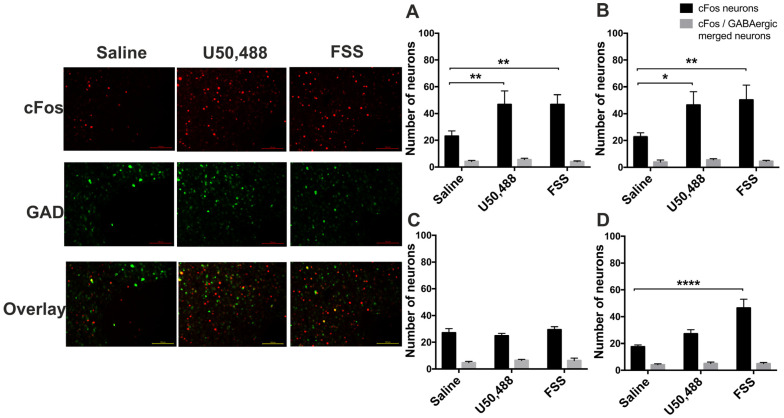
**Activated c-Fos-expressing neurons are non-GABAergic in male and female GAD-GFP transgenic mice.** Representative images of male nucleus accumbens showing immunolabelling for c-Fos and GAD-driven GFP expression following U50,488 (20 mg/kg, ip) treatment or 15 min FSS. Scale bar: 100 μm for all images. Quantification of the number of c-Fos positive cells and those that are double labelled for GFP in the PFCx (**A**,**C**) and NAcc (**B**,**D**) in sections from male (**A**,**B**) and female mice (**C**,**D**). All data are represented as mean ± SEM (n = 6 animals/treatment group).* *p* < 0.05, ** *p* < 0.01, **** *p* < 0.0001. Two-way ANOVA (treatment × neuronal label) with post hoc Dunnett’s test.

To identify the type of neurons where c-Fos expression is enhanced, following acute stress or KOPr activation, we next used GAD-GFP transgenic mice [40]. In these mice, GFP expression is driven by the glutamic acid decarboxylase (*Gad1*) promoter, so GFP is expressed only in GABAergic neurons. We confirmed our earlier findings (from c-Fos-GFP mice) that in male PFCx and NAcc both U50,488 and swim stress increased the number of c-Fos-positive cells (Figure 9A,B), whereas in females neither U50,488 nor FSS increased c-Fos expression in the PFCx (Figure 9C) and FSS, but U50,488 did not increase c-Fos expression in the NAcc (Figure 9D). Quantification of the co-localisation of c-Fos and GAD-GFP-expressing neurons in the PFCx and NAcc, of both male and female mice, revealed that there is very little overlap (Figure 9). For example, in PFCx of male mice the number of c-Fos-positive neurons in the saline treatment group was 23 ± 4, rising to 47 ± 8 in the acute stress treatment group. However, the number of neurons dual-labelled for both c-Fos and GAD-driven GFP was 4 ± 1 and 6 ± 1 in control and treatment groups respectively. Thus only 18% and 12% of neurons were labelled for both c-Fos and GAD-driven GFP, with no recruitment of GAD-positive cells following acute stress. Qualitatively similar findings were observed in female mice and also in the NAcc, suggesting that in both male and female mice, in both the PFCx and NAcc, the cells expressing c-Fos are largely non-GABAergic neurons.

## 3. Discussion

We showed sex-dependent and brain region-dependent increases in c-Fos expression following systemic administration of a KOPr agonist (U50,488) and an acute stressor (FSS), summarized in Table 1. The key findings are as follows: U50,488 increased c-Fos expression in the PFCx and NAcc of male, but not female, mice; FSS similarly increased c-Fos expression in PFCx of male mice only, but responses in NAcc were similar in both sexes. In the CA1 region of the hippocampus and in the amygdala no sex differences were seen in responses to either stimulus. Increases provoked by U50,488, but not FSS, were blocked by the KOPr antagonist norBNI.

As well as quantifying c-Fos expression itself, we examined c-Fos driven GFP expression using c-Fos-GFP transgenic mice. The use of two different antibodies (to c-Fos itself and to GFP) to quantify increases in c-Fos expression validated our overall findings, with comparable regional patterns of activation achieved with both markers (Table 1). c-Fos-GFP transgenic mice also offer the possibility of looking at temporal changes in c-Fos expression. As the half-life of c-Fos is much shorter than that of GFP [36,37,41], if single neurons express GFP, but not c-Fos, that may mean that c-Fos expression occurred at an early stage. Although only a single time-point was used in this study, no significant differences were seen between c-Fos expression or c-Fos-driven GFP expression. The intensity of c-Fos and c-Fos-driven GFP immunofluorescence was essentially the same across all treatment groups, and the majority of individual neurons were positive for both c-Fos and GFP. Changing the endpoint of the experiment, beyond the 2 h window used here, may allow the identification of any temporal pattern of expression. In this study, in every treatment group where c-Fos expression was increased, c-Fos-driven GFP expression was also increased, adding robustness to our observations.

### 3.1. Sex Differences following Systemic KOPr Agonist Administration

Our data demonstrate sex-dependent c-Fos expression in PFCx and NAcc following U50,488 treatment. Blockade by norBNI indicates that these are KOPr-mediated responses that could directly result from different expression levels of KOPr in different brain regions between male and female mice. To our knowledge, there have been no previous studies examining sex differences in KOPr expression in male and female mice, but sex differences are consistent with previous evidence in guinea pigs [42] and humans [43] for brain region-specific sex-dependent differences in KOPr expression. The effects on c-Fos expression reported here could also reflect sex-dependent differences in KOPr signalling, as shown in previous studies [28,42].

Alternatively, the sex-dependent changes in c-Fos expression could be due to more complex network effects, as systemic administration of KOPr agonists can reduce extracellular dopamine, glutamate and GABA release in both the PFCx and NAcc [44,45,46,47,48,49]. It is noteworthy that these studies were only performed in male animals. Although we demonstrated that KOPr- and stress-induced c-Fos expression takes place largely in neurons, not glial cells, KOPrs are expressed in glial cells, particularly astrocytes [50,51,52,53] and their activation could influence neuronal c-Fos expression. Sex-dependence of astrocytic KOPr expression and function has not been studied but it is possible that it may contribute to the sex-dependent neuronal c-Fos expression shown in this study.

### 3.2. Sex Differences following an Acute In Vivo Stressor

We also studied the effects of an acute in vivo stressor. FSS increased c-Fos expression in the NAcc and BLA in both male and female mice, and in the PFCx in male mice only, although baseline levels of c-Fos expression in PFCx after saline injections were higher in female mice compared with male mice. It is possible that the increased saline–saline control values in females (Figure 2) compared to males (Figure 1) reflect increased sensitivity to injection stress that masks any effect of U50,488. No apparent differences in control c-Fos expression were seen in other brain regions. These findings extend previous studies that acute stressors can increase c-Fos expression in various brain regions, including PFCx and NAcc, in male rodents [54,55,56]; these studies did not consider sex differences.

In contrast to its effects on U50,488-evoked responses, norBNI did not prevent stress-induced c-Fos activation (except in NAcc of male mice), although numerous studies have demonstrated a key role for KOPr activation in stress-related responses (eg. [14]). A key difference from U50,488 administration is that dynorphin is the endogenous KOPr agonist released in response to stress. Dynorphin is highly efficacious, with effects seen at very low receptor occupancy [57]. The dose of norBNI used, 10 mg/kg, was previously reported to significantly reduce FSS-induced immobility in male C57Bl/6 mice when tested on day 2 (24 h after dosing [14]), consistent with dose and timescale in the present experiments, but it is possible that the dose of norBNI and/or temporal factors were sub-optimal for countering stress-induced dynorphin effects. Moreover, stress is complex and multifactored, involving various neuroactive substances and their targets, in addition to dynorphin and KOPr [58], therefore the residual c-Fos expression induced by an acute stressor in the presence of nor-BNI may be KOPr-independent.

### 3.3. Functional Role of Sex-Dependent c-Fos Expression following KOPr Agonist Administration and In Vivo Stress

Many rodent studies have identified sex-dependent behavioural consequences of KOPr activation and acute stress (reviewed in [25]). In some studies, this effect is oestrus cycle dependent [29], but independent in others [30,59], and a recent study demonstrated a role for the oestrus cycle in analgesic responses, but not in dysphoric responses [28]. Future studies could explore any role of the oestrus cycle in the sex-dependent c-Fos expression patterns demonstrated here, and how sex-dependent increases in cFos, also demonstrated here, translate into behavioural responses. c-Fos is unlikely to be the only factor switched on by KOPr activation and acute stress and future studies could also examine if other immediate early genes are affected in a similar way.

Previous studies in rats showed that acute stress increased c-Fos expression in the NAcc region, that correlated with both the behavioural effect of the stress and dynorphin A expression [60]. Newton et al. [61] showed that local administration of KOPr antagonists in the NAcc region inhibited the pro-depressive-like effects of footshock stress. However, both studies only used male rats, whereas the present study revealed significant sex differences in the activation of the NAcc region. Understanding the functional consequences of KOPr- and stress-induced c-Fos expression in different brain regions, and in different sexes, requires identification of the precise neurons and pathways where c-Fos was expressed—both neuronal type, and projection sites. First, it is unknown whether the neurons where c-Fos expression was increased in this study also express KOPrs. Although c-Fos is generally thought of as a marker of neuronal activation, its expression can also be increased by inhibitory receptors (such as KOPrs) through ERK signalling [62,63]. Conversely, activation of KOPrs could alter network excitability and affect cFos expression on downstream neurons. Although KOPr-expressing neurons are diverse [64], in cortical and hippocampal regions, they are widely expressed on GABAergic interneurons [65]. We performed initial experiments to examine whether neurons expressing c-Fos following KOPr activation or acute stress, in the PFCx and NAcc, are GABAergic neurons using GAD-GFP mice. Only a very small proportion (<5%) of c-Fos-expressing neurons were GABAergic, and c-Fos expression in GABAergic neurons did not increase in response to KOPr agonist or acute in vivo stressor. Therefore, c-Fos expression following these treatments occurs in non-GABAergic neurons.

### 3.4. Conclusions

The finding presented is that there is sex-dependent c-Fos expression following systemic KOPr agonist administration, while an in vivo stressor may help explain the sexual dymorphism in behavioural responses to both KOPr activation and in vivo stress. Our studies highlight sex-dependent effects in the PFCx and NAcc, with activation of non-GABAergic neurons. These brain regions are known to be involved in motivational behaviours and decision-making [66], and likely play key roles in mediating differential behavioural responses in males and females.

## 4. Materials and Methods

### 4.1. Animals

Adult (9–13 weeks old) male (25–30 g) and female (20–25 g) C57BL/6 c-Fos-GFP transgenic mice [41] or GAD-GFP knock in mice [40] were used throughout these experiments. C-Fos-GFP mice were initially purchased from Jackson Laboratories (Bar Harbor, ME, USA) and express a fusion gene consisting of the murine FBJ osteosarcoma oncogene (*Fos*) and enhanced green fluorescence protein (GFP). GAD-GFP knock in mice express GFP in endogenous glutamic acid decarboxylase (GAD) gene promoter-controlled GABAergic neurons and were a kind gift from Dimitri Kullman (UCL, UK) and rederived by Charles River (Margate, UK). All animals were bred in-house at the University of Bath. At weaning, mice were housed as mixed litter groups of 2–5 in polysulfone cages (35 × 20 × 15 cm), cages with woodchip bedding (Datesand, Manchester, UK) and paper nesting material (Lillico/LBS Biotechnology, Horley, UK). Mice were housed under a 12 h light–dark cycle (lights on at 07:00 h) at 20 ± 2 °C and 50–60% humidity with water and food supplied ad libitum. Mice were assigned randomly to treatment groups and all experiments were performed during the light phase. All mice were handled and weighed one day before stress procedures or drug treatment. All experiments were performed in accordance with the UK Home Office guidelines, including local ethical review, and the Animal (Scientific Procedures) Act 1986/ EU Directive 2010/63/EU.

### 4.2. Drugs

The selective KOPr agonist, U50,488 (20 mg/kg) and the selective KOPr antagonist, norbinaltorphimine (norBNI) (10 mg/kg) were purchased from Tocris Bioscience (Biotechne, Abingdon, UK). All drugs were dissolved in 0.9% *w*/*v* saline (Hameln Pharmaceuticals, Gloucester, UK) and administered intraperitoneally (i.p.) at a volume of 10 mL/kg. NorBNI is identified to produce slow onset of activity (peak at 24 h) and long-lasting effects (up to 3–4 weeks) [67]. Therefore, norBNI was administered 24 h before stress or U50,488 treatment. In any given experiment, all animals received the same number of injections to control for injection stress.

### 4.3. Acute Forced Swim Stress (FSS)

Forced swimming has been used as an acute stressor for a single exposure up to 15 min or repeated exposure to forced swim in mice [68] and rats [69]. To induce stress, mice were gently placed in a glass beaker (height 34 cm, diameter 22 cm) with water (25 ± 1 °C) filled to approximately two thirds of the total beaker volume (height 23 cm). Each mouse was allowed to swim for 15 min and observed throughout to monitor welfare. After 15 min, the mice were removed from the water and gently dried with paper towels. Mice were placed into a holding cage for 10 min, standing on a heated blanket, to reduce the risk of hypothermia before returning to the home cage. Between each mouse, the water was emptied, the beaker cleaned with 70% ethanol, rinsed, and replaced with fresh water.

### 4.4. Immunohistochemistry

Mice were treated with either a single injection of selective KOPr agonist, U50,488 (20 mg/kg, i.p.) or acute in vivo stressor (FSS, 15 min). Both procedures produced a significant increase in plasma corticosterone levels (Supplementary Figure S1). A high dose of U50,488 was used to ensure activation of all KOPr [70] and caused a significant reduction in locomotor behaviour in both male and female mice (Supplementary Figure S1). After 2 h in the home cage, mice were anaesthetised with pentobarbital (100 mg/kg, i.p.) and perfused intracardially with 20 mL of 0.01 M phosphate buffered saline (PBS), pH 7.4, followed by 20 mL of 4% paraformaldehyde (PFA), pH 7.4 in 0.01 M PBS. The brain was removed and placed in a specimen bottle and post-fixed and stored in 4% PFA at 4 °C overnight. The brain was then permeated with 30% sucrose in PBS at 4 °C for at least 16 h before immunohistochemistry. The brain tissues were cut into coronal sections (40 μm) on a vibratome (Leica, Germany), including prefrontal cortex (PFCx) (Bregma +2.22 mm), nucleus accumbens (NAcc) (Bregma +1.34 mm), dorsal hippocampus (Bregma −1.94 mm), and amygdala (Bregma −1.94 mm). All washes were performed in PBS at room temperature. Free-floating sections were rinsed and incubated in blocking solution (0.01 M PBS containing 0.1% Triton X-100 and 5% donkey serum) for 2 h at room temperature. Subsequently, sections were incubated overnight at 4 °C with primary antibodies diluted in 0.01 M PBS containing 0.1% Triton X-100 and 2.5% donkey serum: rabbit anti-c-Fos antibody (1:500, Cell Signaling Technology, #2250, Danvers, MA, USA) or chicken anti-GFP antibody (1:1000, Abcam (Cambridge, UK), ab13970) or for the glia cell marker anti-GFAP (glial fibrillary acidic protein; (1:1000, Abcam, ab4674, chicken anti-GFAP) or for the neuronal cell marker anti-NeuN (1:500, Merck (Darmstadt, Germany), ABN91, chicken anti-NeuN). Tissue sections were rinsed with PBS and then incubated with the appropriate secondary antibodies diluted in 0.01 M PBS containing 0.1% Triton X-100 and 2.5% donkey serum for 2 h at room temperature: Alexa Fluor 568 goat anti-rabbit antibody (1:500, Thermo Fisher Scientific (Horsham, UK), A11011); Alexa Fluor 488 goat anti-chicken antibody (1:500, Abcam, ab150169). After washing for 2 h, sections were mounted onto microscope slides in DAPI-containing Vectashield (Vector Laboratories, Newark, CA, USA) and viewed using a Leica (Milton Keynes, UK) DMI4000B inverted wide-field fluorescent microscope at 200× magnification.

### 4.5. Quantification and Statistical Analysis

For each mouse, one coronal section (40 μm) was imaged for each brain region. Control and treatment groups were processed in parallel and analysed the same way. All analysis was performed with the experimenter masked to treatment. Observed fluorescent signal was measured by defining a region of interest and measuring total fluorescence/pixel. Background labelling was assessed in a non-labelled part of the brain section using a similar region of interest approach and subtracted from the total fluorescence/pixel measurement for each section. Reference to a mouse brain atlas [38] and light field images were used to identify landmarks and define regions of interest: nucleus accumbens core, prefrontal cortex (prelimbic areas I–III), amygdala (central nucleus was distinguished from basolateral amygdala), and hippocampus (CA1 and dentate gyrus regions distinguished). All labelled cells in the region of interest were confirmed as cellular using DAPI staining. All fluorescent signal intensities were directly measured in ImageJ v1.50 software. For co-labelling c-Fos/GFAP and c-Fos/NeuN experiments, the total c-Fos-positive neurons were manually counted and those co-expressing c-Fos/GFAP or c-Fos/NeuN identified and expressed as a percentage of the total number of c-Fos-positive neurons.

All treatment groups shown in an individual graph were processed in parallel. A single microscope slide had sections from each treatment group to overcome any day-to-day variability in antibody labelling and microscopy in the quantification and analysis.

Normalized fluorescent signals in each region of interest were analysed using GraphPad Prism (version 9.3.1) with one-way analysis of variance (ANOVA) with ‘Treatment’ as a factor followed by post hoc Sidak’s test. Comparisons of c-Fos and GAD-driven GFP fluorescence intensity were analysed with two-way ANOVA (treatment × neuronal marker) with post hoc Dunnett’s test. All data are presented as mean ± standard error of the mean (S.E.M) and significance levels were *p* < 0.05.

## Figures and Tables

**Table 1:** **Summary of c-Fos and c-Fos-induced GFP (c-Fos-GFP) expression in different brain regions in adult male and female c-Fos-GFP transgenic mice.** Mice were given either the KOPr agonist U50,488 (20 mg/kg) or FSS in the presence and absence of the KOPr antagonist norBNI (10 mg/kg). Prefrontal cortex (PFCx), nucleus accumbens (NAc), CA1 and dentate gyrus region of hippocampus (CA1, DG), central amygdala (CeA), basolateral amygdala (BLA). ↑, * <0.05; ↑↑, ** <0.01; ↑↑↑ <0.001; ns = not significant.

Male	U50,488				Forced Swim Stress			
	c-Fos		c-Fos-GFP		c-Fos		c-Fos-GFP	
	Saline	norBNI	Saline	norBNI	Saline	norBNI	Saline	norBNI
PFCx	*↑* *↑*	**	*↑*	*	*↑* *↑*	ns	*↑* *↑*	ns
NAc	*↑* *↑*	**	*↑*	**	*↑* *↑*	*	*↑* *↑*	ns
CA1	*↑*	*	*↑* *↑*	**	ns	ns	ns	ns
DG	ns	ns	ns	Ns	ns	ns	ns	ns
CeA	*↑*	*	*↑*	*	ns	ns	ns	ns
BLA	*↑*	*	*↑* *↑*	**	*↑*	ns	*↑*	ns
**Female**	**U50,488**				**Forced swim stress**			
	**c-Fos**		**c-Fos-GFP**		**c-Fos**		**c-Fos-GFP**	
	**Saline**	**norBNI**	**Saline**	**norBNI**	**Saline**	**norBNI**	**Saline**	**norBNI**
PFCx	ns	ns	ns	ns	ns	ns	ns	ns
NAc	ns	ns	ns	ns	*↑* *↑*	ns	*↑*	ns
CA1	*↑* *↑* *↑*	**	ns	ns	ns	ns	ns	ns
DG	ns	ns	ns	ns	ns	ns	ns	ns
CeA	*↑*	*	*↑* *↑*	*	ns	ns	ns	ns
BLA	*↑* *↑* *↑*	**	*↑* *↑*	*	*↑* *↑* *↑*	ns	*↑*	ns

## Data Availability

Supporting data are available on request to the corresponding author.

## Supplementary Materials

The following supporting information can be downloaded at: https://www.mdpi.com/article/10.3390/ijms242015098/s1 [71].
