# Supplementary material for: Sex Differences in Brain Region-Specific Activation of c-Fos following Kappa Opioid Receptor Stimulation or Acute Stress in Mice
Source: Int J Mol Sci. 2023 Oct 11;24(20):15098. doi: 10.3390/ijms242015098 (PMC10606335; doi:10.3390/ijms242015098)

Supplementary Materials

Figure S1. Effects of forced swim stress (FSS) (A,C) and U50,488 (20mg/kg; B,D) on plasma corticosterone levels (A-D) and open field locomotor behaviour (E) following administration of U50,488 in female (B) and male (D) cFos-GFP transgenic mice. (A-D) Blood samples (~40 μl) were collected from the lateral tail vein using the tail incision method [71]. Samples were collected at baseline (24 hours prior to FSS or U50,488 treatment) and test samples collected the next day 10 minutes after FSS and 30 minutes after administration of U50,488 (20mg/kg). Control groups were either non-stressed (for FSS) or received saline injections, with all animals receiving the same number of injections. Nor-BNI (10 mg/kg) or saline was administered immediately after baseline blood samples were collected: 24h prior to FSS or U50,488 administration (N=5 per group, two-way ANOVA with Sidak's test ***$P < 0.001$). (E) Whole distance travelled in the open field (cm) 30 minutes post-injection (10 ml/kg, ip) detected by photobeam breaks during a 10 min test at low lighting level (50 lux) (N=6-7 per group). Experiments were completed between 10:00 and 15:00h in the light cycle. (N=6 per group, one-way ANOVA with Dunnett's test ***$P <$ 0.001).

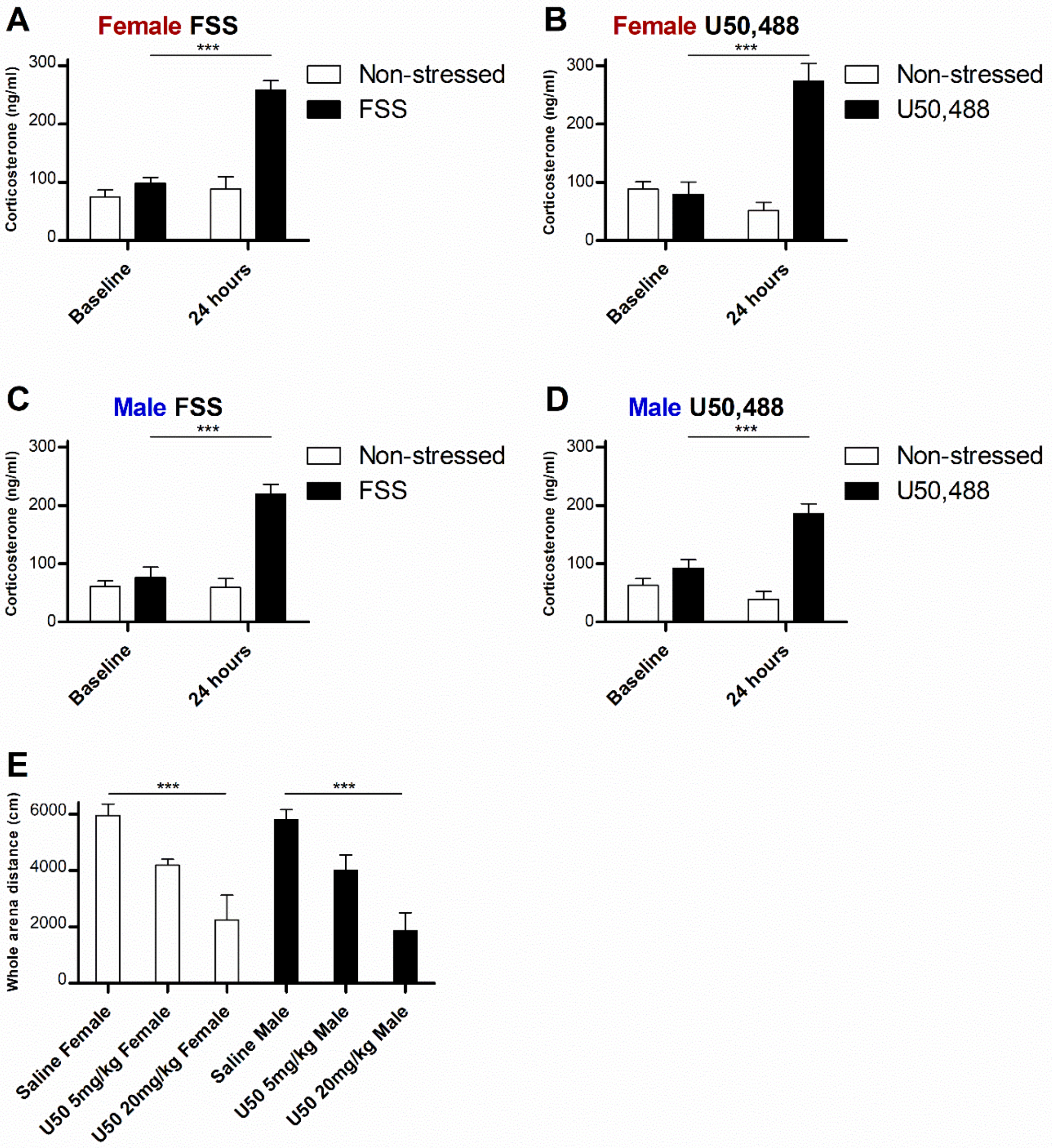
A
Female FSS
***
300
200
100
0
Corticosterone (ng/ml)
Non-stressed
FSS
Baseline
24 hours
B
Female U50,488
***
Corticosterone (ng/ml)
Non-stressed
U50,488
Baseline
24 hours
C
Male FSS
***
Corticosterone (ng/ml)
Non-stressed
FSS
Baseline
24 hours
D
Male U50,488
***
Corticosterone (ng/ml)
Non-stressed
U50,488
Baseline
24 hours
E
***
***
6000
4000
2000
0
Whole arena distance (cm)
Saline Female
U50 5mg/kg Female
U50 20mg/kg Female
Saline Male
U50 5mg/kg Male
U50 20mg/kg Male

Figure S2. The effect of U50,488 and forced swim stress (FSS) on c-Fos-driven GFP expression in the nucleus accumbens of male and female c-Fos-GFP transgenic mice. Representative images of male (A-D) and female (F-J) nucleus accumbens showing immunolabelling for c-Fos-GFP following treatment with saline/saline (A, F), saline/U50,488 (B, G), norBNI/saline (C, H), norBNI/ U50,488 (D, J). Scale bar: 100μm for all images. Quantification of immunolabelling for c-Fos-GFP in male (E) and female (K) sections (Fig 2E, $F(3,20) = 6.627$, $P = 0.003$; Fig 2K $F(3,20) = 4.975$, $P = 0.336$). Representative images of male (L-O) and female (Q-T) nucleus accumbens showing immunolabelling for c-Fos-GFP following acute stress treatment: saline/no stress (NS) controls (L, Q), saline/FSS (M, R), norBNI/ no stress (N, S), norBNI/ FSS (O, T). Quantification of immunolabelling for c-Fos-GFP in male (P) and female (U) sections, (Fig 2P, $F(3,20) = 8.512$, $P = 0.002$; Fig 2U $F(3,20) = 4.737$, $P = 0.012$). All data are represented as mean ± SEM (n=6 animals/treatment group). One-way ANOVA with post-hoc Sidak's test $*P < 0.05$, $**P < 0.01$.

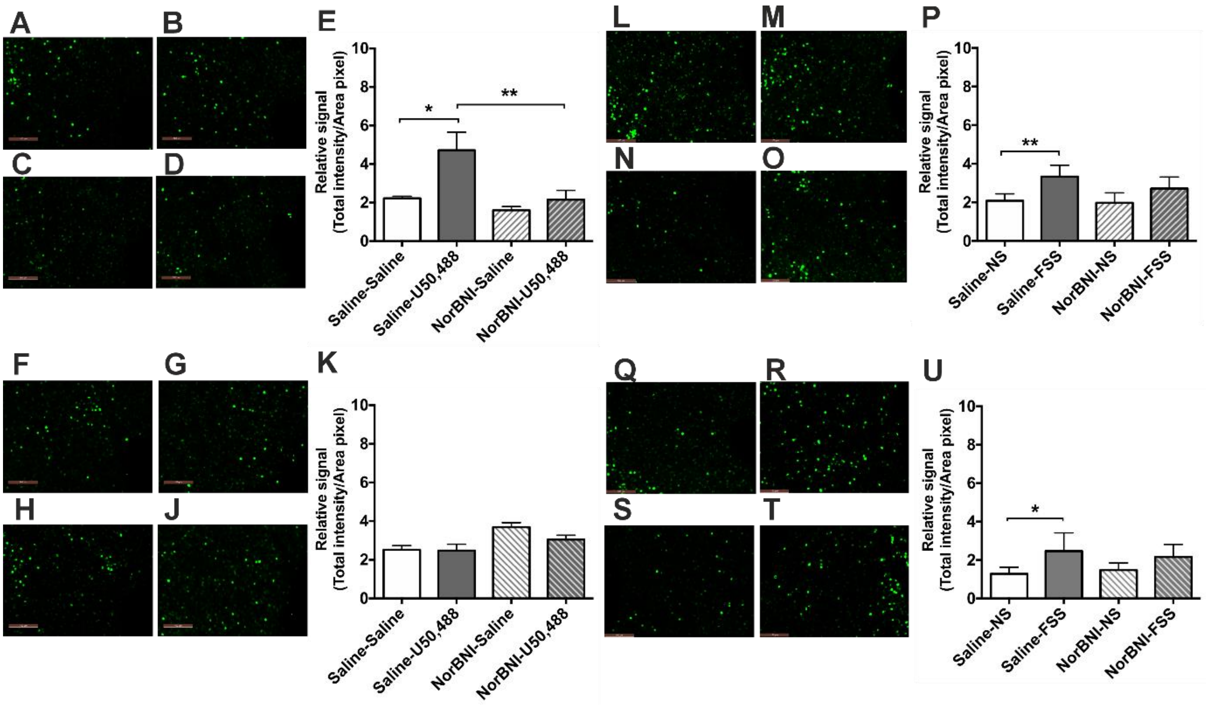

Figure S3. The effect of U50,488 and forced swim stress (FSS) on c-Fos-driven GFP expression in the hippocampal CA1 region of male and female c-Fos-GFP transgenic mice. Representative images of male (A-D) and female (F-J) CA1 region showing immunolabelling for c-Fos-GFP following treatment with saline/saline (A, F), saline/U50,488 (B, G), norBNI/saline (C, H), norBNI/ U50,488 (D, J). Scale bar: 100µm for all images. Quantification of immunolabelling for c-Fos-GFP in male (E) and female (K) sections (Fig 3E, $F(3,20) = 8.155$, $P = 0.001$; Fig 3K, $F(3,20) = 3.368$, $P = 0.069$). Representative images of male (L-O) and female (Q-T) hippocampal CA1 region showing immunolabelling for c-Fos-GFP following acute stress treatment: saline/no stress (NS) controls (L, Q), saline/FSS (M, R), norBNI/ no stress (N, S), norBNI/ FSS (O, T). Quantification of immunolabelling for c-Fos-GFP in male (P) and female (U) sections (Fig 3P, $F(3,20) = 2.829$, $P = 0.065$; Fig 3U, $F(3,20) = 9.693$, $P = 0.275$). All data are represented as mean ± SEM (n=6 animals/treatment group). One-way ANOVA with post-hoc Sidak's test $^{**}P < 0.01$.

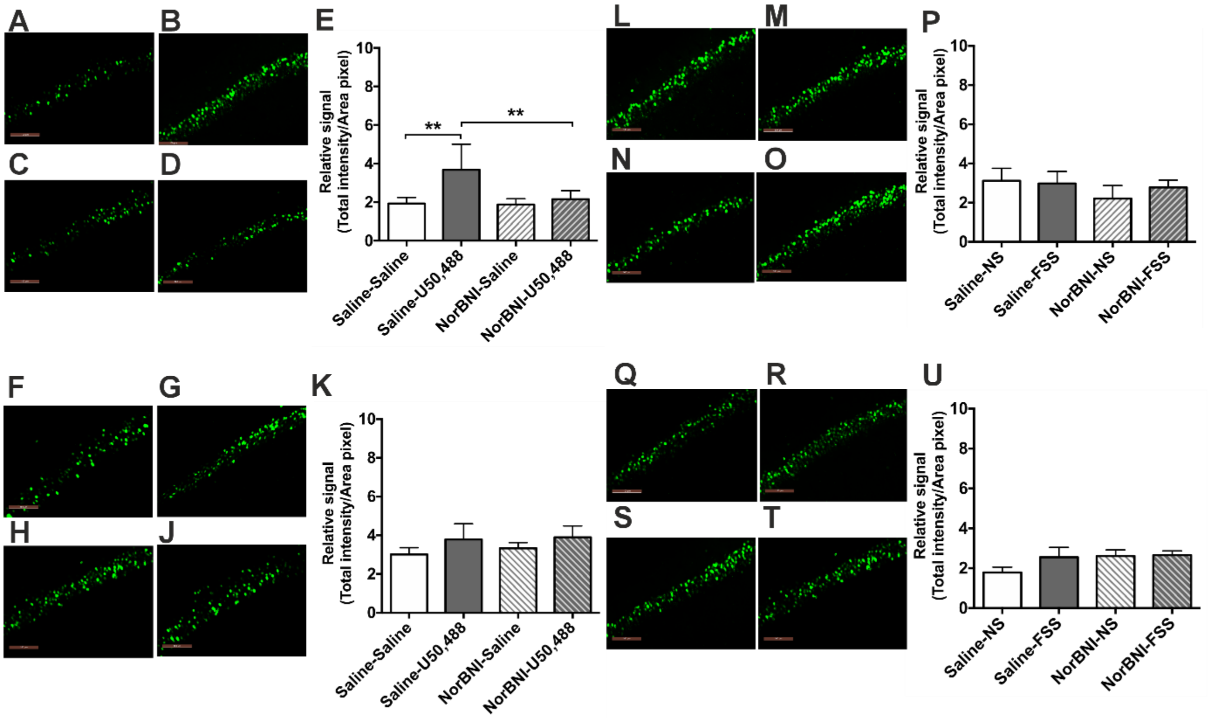

Figure S4. The effect of U50,488 and forced swim stress (FSS) on c-Fos expression in the dentate gyrus of the hippocampus of male and female c-Fos-GFP transgenic mice. Representative images of male (A-D) and female (F-J) dentate gyrus region showing immunolabelling for c-Fos following treatment with saline/saline (A, F), saline/U50,488 (B, G), norBNI/saline (C, H), norBNI/ U50,488 (D, J). Scale bar: 100μm for all images. Quantification of immunolabelling for c-Fos in male (E) and female (K) sections (Fig 4E, $F(3,20) = 4.980$, $P = 0.226$; Fig 4K, $F(3,20) = 3.786$, $P = 0.432$). Representative images of male (L-O) and female (Q-T) hippocampal dentate gyrus region showing immunolabelling for c-Fos following acute stress treatment: saline/no stress (NS) controls (L, Q), saline/FSS (M, R), norBNI/ no stress (N, S), norBNI/ FSS (O, T). Quantification of immunolabelling for c-Fos-GFP in male (P) and female (U) sections (Fig 4P, $F(3,20) = 13.29$, $P = 0.584$; Fig 4U, $F(3,20) = 4.095$, $P = 0.340$). All data are represented as mean ± SEM (n=6 animals/treatment group).

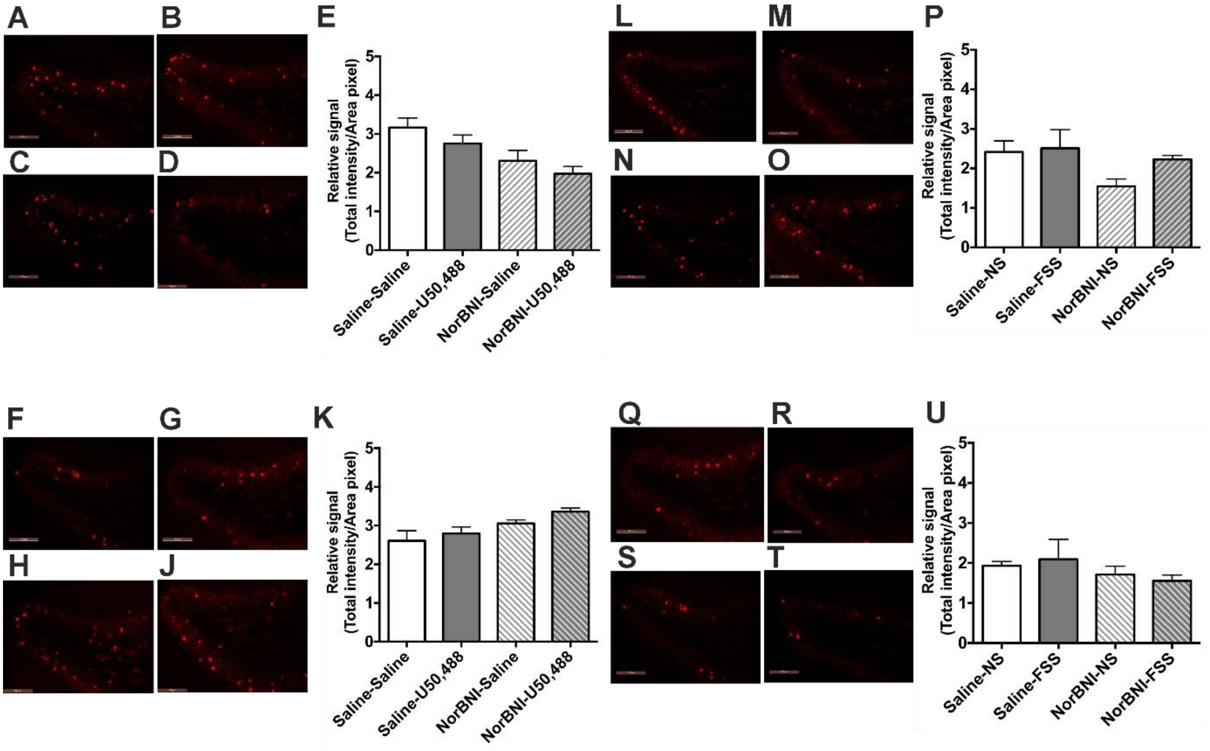

Figure S5. The effect of U50,488 and forced swim stress (FSS) on c-Fos-driven GFP expression in the dentate gyrus of the hippocampus of male and female c-Fos-GFP transgenic mice. Representative images of male (A-D) and female (F-J) dentate gyrus region showing immunolabelling for c-Fos-GFP following treatment with saline/saline (A, F), saline/U50,488 (B, G), norBNI/saline (C, H), norBNI/ U50,488 (D, J). Scale bar: 100μm for all images. Quantification of immunolabelling for c-Fos-GFP in male (E) and female (K) sections (Fig 5E, $F(3,20) = 1.382$, $P = 0.277$; Fig 5K, $F(3,20) = 4.382$, $P = 0.137$). Representative images of male (L-O) and female (Q-T) hippocampal dentate gyrus region showing immunolabelling for c-Fos-GFP following acute stress treatment: saline/no stress (NS) controls (L, Q), saline/FSS (M, R), norBNI/ no stress (N, S), norBNI/ FSS (O, T). Quantification of immunolabelling for c-Fos-GFP in male (P) and female (U) sections (Fig 5P, $F(3,20) = 1.382$, $P = 0.277$; Fig 5U, $F(3,20) = 4.382$, $P = 0.137$). All data are represented as mean ± SEM (n=6 animals/treatment group).

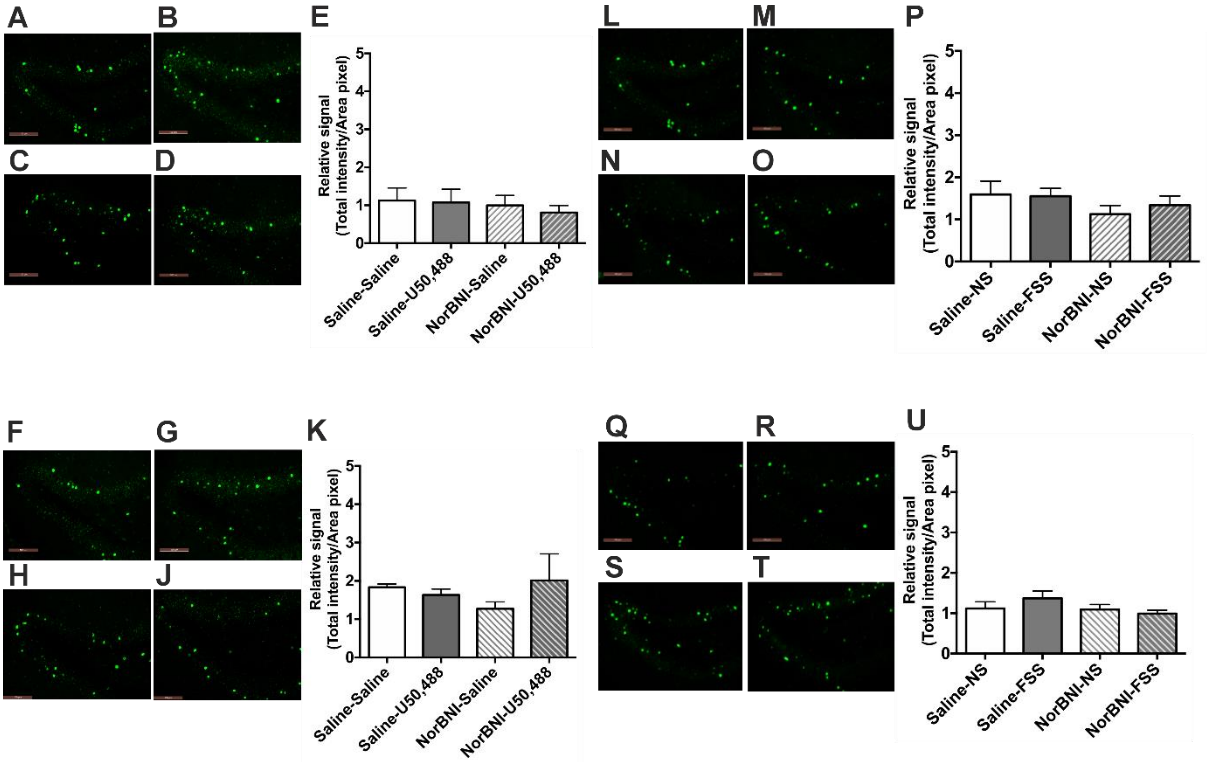

Figure S6. The effect of U50,488 and forced swim stress (FSS) on c-Fos-driven GFP expression in the central nucleus of the amygdala of male and female c-Fos-GFP transgenic mice. Representative images of male (A-D) and female (F-J) central nucleus region showing immunolabelling for c-Fos-GFP following treatment with saline/saline (A, F), saline/U50,488 (B, G), norBNI/saline (C, H), norBNI/ U50,488 (D, J). Scale bar: 100μm for all images. Quantification of immunolabelling for c-Fos-GFP in male (E) and female (K) sections (Fig 6E, $F(3,20) = 4.543$, $P = 0.014$; Fig 6K, $F(3,20) = 5.118$, $P = 0.009$). Representative images of male (L-O) and female (Q-T) amygdala central nucleus region showing immunolabelling for c-Fos-GFP following acute stress treatment: saline/no stress (NS) controls (L, Q), saline/FSS (M, R), norBNI/ no stress (N, S), norBNI/ FSS (O, T). Quantification of immunolabelling for c-Fos-GFP in male (P) and female (U) sections (Fig 6P, $F(3,20) = 4.572$, $P = 0.988$; Fig 6U, $F(3,20) = 1.651$, $P = 0.176$). All data are represented as mean ± SEM (n=6 animals/treatment group). One-way ANOVA with post-hoc Sidak's test $*P < 0.05$, $**P < 0.01$.

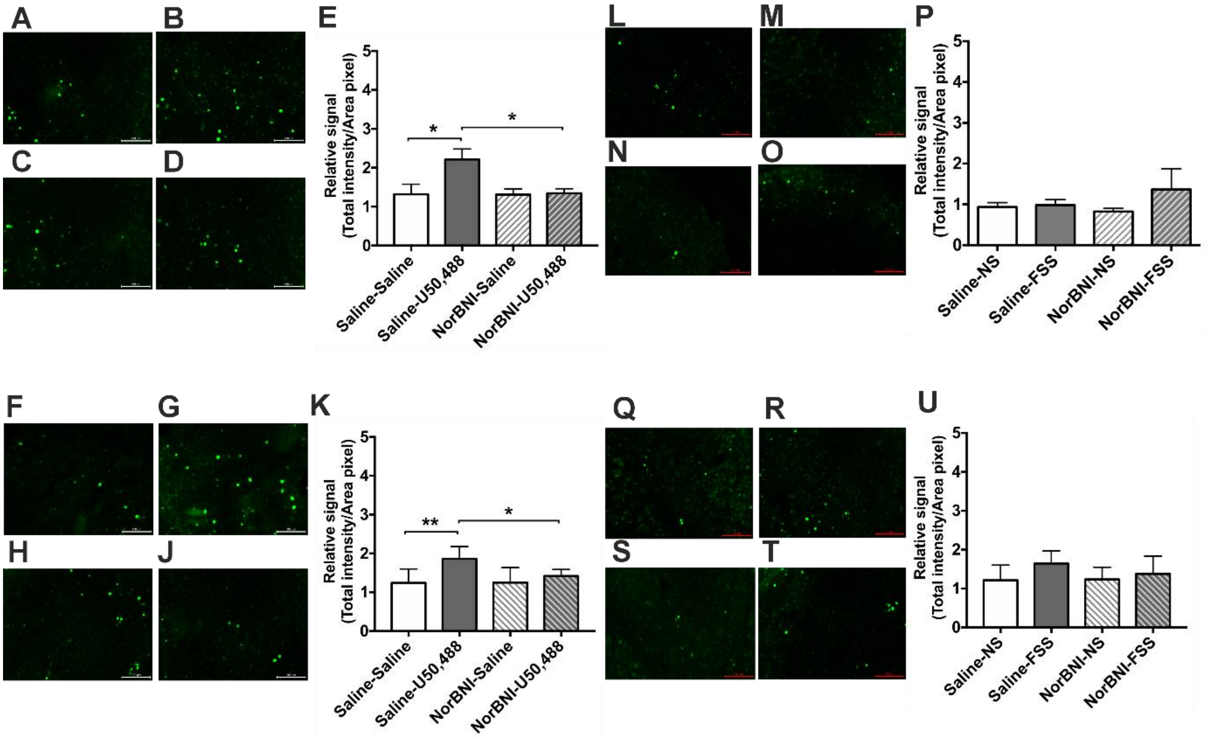

Figure S7. The effect of U50,488 and forced swim stress (FSS) on c-Fos expression in the basolateral nucleus of the amygdala of male and female c-Fos-GFP transgenic mice. Representative images of male (A-D) and female (F-J) basolateral nucleus region showing immunolabelling for c-Fos following treatment with saline/saline (A, F), saline/U50,488 (B, G), norBNI/saline (C, H), norBNI/ U50,488 (D, J). Scale bar: 100μm for all images. Quantification of immunolabelling for c-Fos in male (E) and female (K) sections (Fig 7E, $F(3,20) = 7.164$, $P = 0.001$; Fig 7K, $F(3,20) = 10.56$, $P = 0.0002$). Representative images of male (L-O) and female (Q-T) amygdala basolateral nucleus region showing immunolabelling for c-Fos following acute stress treatment: saline/no stress (NS) controls (L, Q), saline/FSS (M, R), norBNI/ no stress (N, S), norBNI/ FSS (O, T). Quantification of immunolabelling for c-Fos-GFP in male (P) and female (U) sections (Fig 7E, $F(3,20) = 6.403$, $P = 0.016$; Fig 7K, $F(3,20) = 17.60$, $P = 0.0001$). All data are represented as mean ± SEM (n=6 animals/treatment group). One-way ANOVA with post-hoc Sidak's test $*P < 0.05$, $**P < 0.01$, $***P < 0.001$

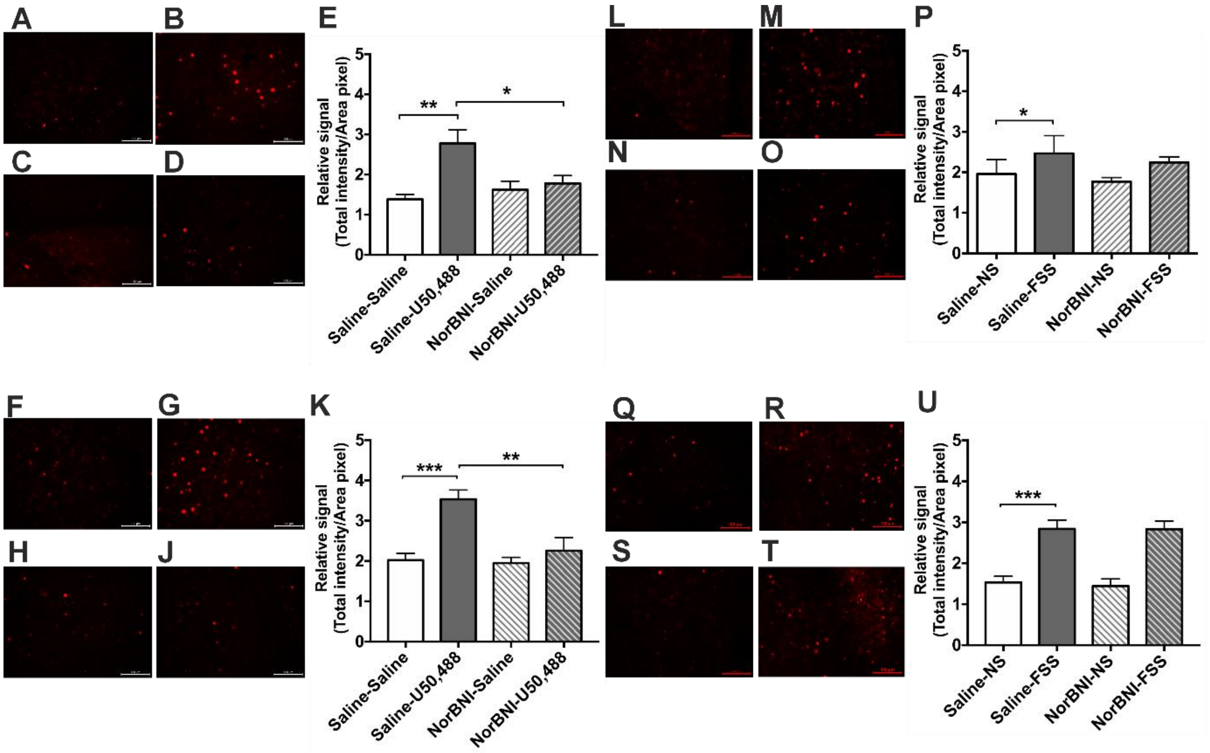

Figure S8. The effect of U50,488 and forced swim stress (FSS) on c-Fos-driven GFP expression in the basolateral nucleus of the amygdala of male and female c-Fos-GFP transgenic mice. Representative images of male (A-D) and female (F-J) basolateral nucleus region showing immunolabelling for c-Fos-GFP following treatment with saline/saline (A, F), saline/U50,488 (B, G), norBNI/saline (C, H), norBNI/ U50,488 (D, J). Scale bar: 100μm for all images. Quantification of immunolabelling for c-Fos-GFP in male (E) and female (K) sections (Fig 8E, $F(3,20) = 7.433$, $P = 0.002$; Fig 8K, $F(3,20) = 9.119$, $P = 0.002$). Representative images of male (L-O) and female (Q-T) amygdala basolateral nucleus region showing immunolabelling for c-Fos-GFP following treatment with saline/no stress (NS) controls (L, Q), saline/FSS (M, R), norBNI/ no stress (N, S), norBNI/ FSS (O, T). Quantification of immunolabelling for c-Fos-GFP in male (P) and female (U) sections (Fig 8P, $F(3,20) = 9.343$, $P = 0.012$; Fig 8U, $F(3,20) = 6.321$, $P = 0.011$). All data are represented as mean ± SEM (n=6 animals/treatment group). One-way ANOVA with post-hoc Sidak's test *$P < 0.05$, **$P < 0.01$.

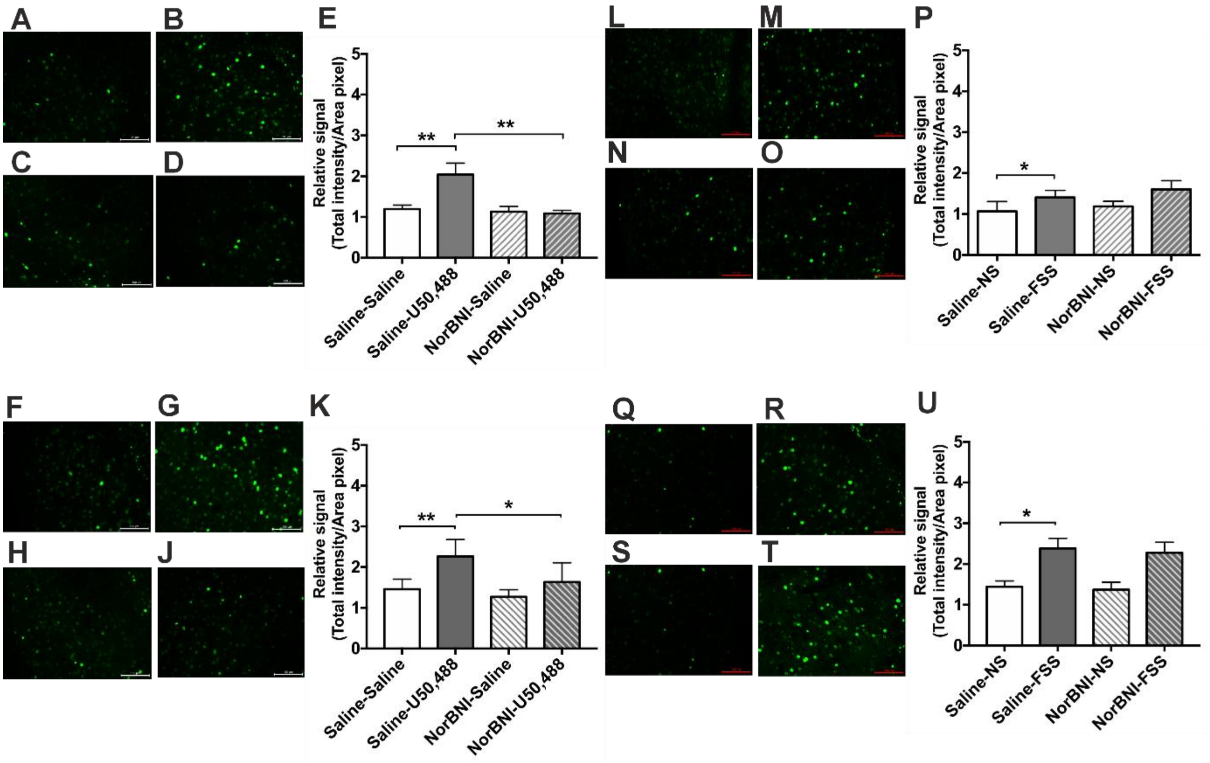

Figure S9. Activated c-Fos-expressing cells in the nucleus accumbens of male and female c-Fos-GFP transgenic mice are predominantly neuronal cells. Following treatment with U50,488 (20 mg/kg) or forced swim stress (FSS, single 15 min swim), quantification of the proportion of c-Fos positive cells that are double labelled for GFAP (A, B, glial cell marker) or for Neu N (C,D, neuronal cell marker) in male (A, C) and female (B, D) sections. Data for each individual sample is shown (O) and the mean (X), n= 2-3 animals/treatment group.

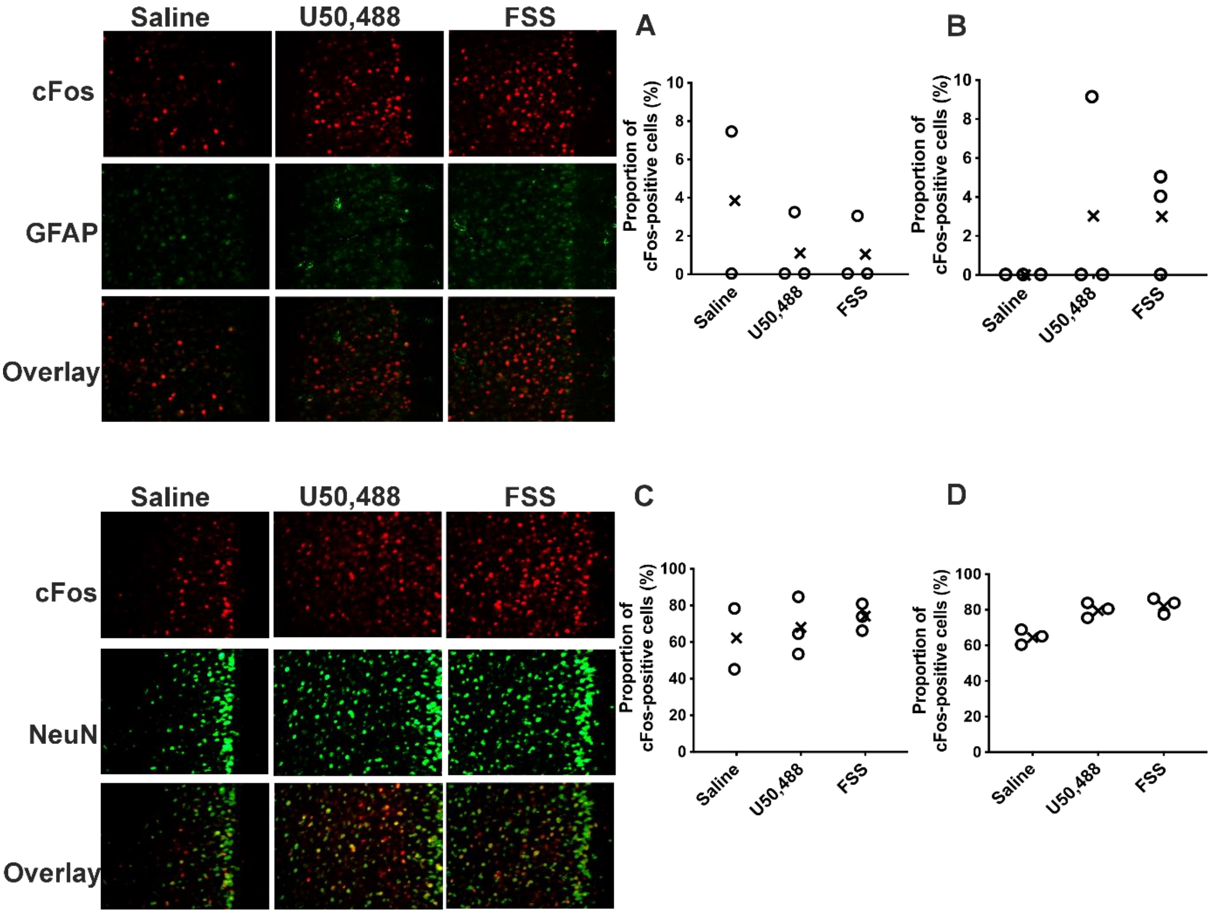

Supplement: Supplementary file 1 [file ijms-24-15098-s001.zip › ijms-2608849-supplementary.pdf]
